# Donovanosis – A rare, sexually transmitted infection that the world has not yet eradicated^؆^

**DOI:** 10.1016/j.abd.2026.501411

**Published:** 2026-06-27

**Authors:** Ana Tereza Orsi, Sanmya Vitoria Bernardino de Oliveira, Luiz Carlos de Lima Ferreira, Antonio Pedro Mendes Schettini, Silvana de Albuquerque Damasceno Ferreira, Sinésio Talhari

**Affiliations:** a Fundação Hospitalar de Dermatologia Tropical e Venereologia Alfredo da Matta, Manaus, AM, Brazil; b Department of Pathology and Forensic Medicine, Faculty of Medicine, Universidade Federal do Amazonas, Manaus, AM, Brazil

Dear Editor,

Donovanosis or granuloma inguinale is a chronic, indolent, and progressive disease with low infectivity, caused by the bacterium *Klebsiella granulomatis*.^1^ Donovanosis transmission occurs predominantly through sexual contact,^2^ although cases have been reported in children and adults without sexual activity through contact with infected adults. There are also reports of patients with extra-genital lesions without concomitant genital lesions, making contamination through feces possible, as a bacterium similar to *Klebsiella granulomatis* has been identified in the feces of a patient with the disease.^1^

It is considered endemic in some tropical and subtropical regions, including parts of Brazil, South Africa, India, and Australia.^3^ In the 1990s, an increase in the number of cases was observed, mainly in South Africa, after the use of mass rapid testing,^1^ suggesting that the low incidence is a consequence of underdiagnosis. Currently, the number of new cases is low, being described only sporadically,^1^ without clear numerical data in Brazil and worldwide.

This article reports a case of donovanosis, diagnosed at a reference center for STIs in the city of Manaus, in the Brazilian Amazonia. The patient, a 21-year-old indigenous woman from the municipality of Tabatinga, state of Amazonas, has cognitive and motor impairment. Her mother reports that, eight months ago, she observed lesions on the vulva and anal region, associated with pain, pruritus, and bleeding. Physical examination revealed multiple coalescent vegetative and ulcerated lesions in the vulvar and anal region, with areas of bleeding, as well as lymphedema of the right lower limb and bilateral adenomegaly (Figs. 1A and 1B; Fig. 1C – lesion after treatment). Family members are unaware of the patient’s any sexual exposure. Serological tests for hepatitis B and C, syphilis, and HIV, performed in December 2023, were negative.

**Fig. 1:**
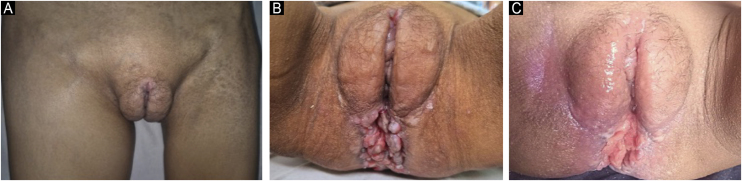
(A) Lymphedema of the right lower limb (June 2024). (B) Vegetative and ulcerated lesions in the perianal region and perineum (June 2024). (C) Regression of lesions after treatment (December 2024).

Donovanosis is characterized by lesions that generally begin as painless papules, developing into nodules, vegetating lesions, or ulcers, which bleed easily and may have aspects similar to carcinoma.^4^ Elephantiasis, associated with ulcerated and cicatricial lesions, as observed in the present case, is relatively frequent in patients with a long course of the disease. In all these patients, serology for HIV, syphilis, hepatitis B and C is recommended. Malignant neoplasms, American cutaneous leishmaniasis, and cutaneous tuberculosis, among other diseases, are among the main differential diagnoses.^5^

In the reported case, the diagnosis was attained by histopathological examination, which revealed an epidermis with acanthosis and marked inflammatory infiltrate in the dermis, consisting of vacuolated lymphocytes and histiocytes (Fig. 2A). Warthin-Starry staining revealed a large number of Donovan bodies in the cytoplasm of histiocytes (Fig. 2B), characteristic of *Klebsiella granulomatis* infection.^6^

**Fig. 2:**
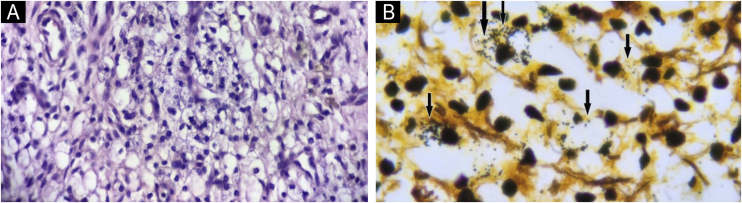
Microscopic examination showing inflammatory infiltrate of vacuolated histiocytes and lymphocytes. (A) Hematoxylin & eosin, ×400. (B) Warthin-Starry. Donovan bodies (arrows; ×1000).

For the treatment of donovanosis, azithromycin is recommended as a first-line drug, given its efficacy and safety. Among the alternative drugs, doxycycline, ciprofloxacin, and sulfamethoxazole-trimethoprim are indicated,^1^, ^3^, ^4^ especially in resistant cases or patients who cannot use azithromycin. In immunosuppressed patients, gentamicin or chloramphenicol is indicated.^1^, ^6^ For the case of the present study, doxycycline was administered at a dose of 100 mg every 12 hours. After 21 days of treatment, partial regression of the lesions and significant regression of the lymphadenopathy were observed.

Despite the relative rarity of donovanosis in our setting, it is believed it is important to emphasize that the diagnosis of donovanosis should always be considered in patients with ulcerated lesions located in the male or female genitalia, perineum, and anorectal region. According to records from Fundação Alfredo da Matta, the last diagnosis of donovanosis occurred in 2007.

## ORCID ID

Ana Tereza Orsi: 0009-0007-7216-7589

Luiz Carlos de Lima Ferreira: 0000-0002-9657-939X

Antonio Pedro Mendes Schettini: 0000-0002-5357-9189

Silvana de Albuquerque Damasceno Ferreira: 0000-0002-8873-0395

Sinésio Talhari: 0000-0001-9753-6706

## Financial support

None declared.

## Authors' contributions

Ana Tereza Orsi: Design and planning of the study; drafting and editing of the manuscript; collection, analysis and interpretation of data; effective participation in research orientation; intellectual participation in the propaedeutic and/or therapeutic conduct of the studied case; critical review of the literature; critical review of the manuscript; approval of the final version of the manuscript.

Sanmya Vitoria Bernardino de Oliveira: Drafting and editing of the manuscript; collection, analysis and interpretation of data; intellectual participation in the diagnosis of the studied case; critical review of the literature; critical review of the manuscript.

Luiz Carlos de Lima Ferreira: Drafting and editing of the manuscript; collection, analysis and interpretation of data; intellectual participation in the diagnosis of the studied case; critical review of the literature; critical review of the manuscript.

Antonio Pedro Mendes Schettini: Drafting and editing of the manuscript; collection, analysis and interpretation of data; intellectual participation in the diagnosis of the studied case; critical review of the literature; critical review of the manuscript.

Silvana de Albuquerque Damasceno Ferreira: Drafting and editing of the manuscript; collection, analysis, and interpretation of data; intellectual participation in the diagnosis of the studied case; critical review of the literature; critical review of the manuscript.

Sinésio Talhari: Drafting and editing of the manuscript; collection, analysis, and interpretation of data; effective participation in research orientation; intellectual participation in the propaedeutic and/or therapeutic conduct of the studied case; critical review of the literature; critical review of the manuscript; approval of the final version of the manuscript.

## Research data availability

Does not apply.

## Conflicts of interest

None declared.

## References

[1] Belda Junior W. (2020). Donovanosis. An Bras Dermatol..

[2] Bezerra S.M.F.M.C., Jardim M.M.L., Silva V.B. (2011). Donovanose. An Bras Dermatol..

[3] Workowski K.A., Bachmann L.H., Chan P.A., Johnston C.M., Muzny C.A., Park I. (2021). Sexually transmitted infections treatment guidelines, 2021. MMWR Recomm Rep..

[4] O’Farrell N., Moi H. (2016). 2016 European guideline on donovanosis. Int J STD AIDS..

[5] Brasil (2018). Ministério da Saúde. Secretaria de Vigilância em Saúde. Protocolo clínico e diretrizes terapêuticas para atenção integral às pessoas com infecções sexualmente transmissíveis (IST) [Internet].

[6] Calonje E., Brenn T., Lazar A. (2012).

